# Increased serum adiponectin predicts improved coronary flow and clinical outcomes in patients with ST‐segment elevation myocardial infarction treated by primary percutaneous coronary intervention

**DOI:** 10.1002/jcla.22864

**Published:** 2019-02-19

**Authors:** Siwen Liang, Hongwei Li, Xuhua Shen, Ruifeng Liu

**Affiliations:** ^1^ Department of Cardiology, Beijing Friendship Hospital Capital Medical University Beijing China

**Keywords:** acute myocardial infarction, adiponectin, major adverse cardiac event, myocardial blood flow, primary percutaneous coronary intervention

## Abstract

**Background:**

Previous studies suggested that adiponectin (APN) could ameliorate ischemia/reperfusion injury and endothelial dysfunction in patients with acute myocardial infarction. However, the relationship between serum APN level and coronary flow after primary percutaneous coronary intervention (PPCI) in patients with ST‐segment elevation myocardial infarction (STEMI) is unclear.

**Methods:**

A total of 144 patients with STEMI treated by PPCI were enrolled and divided into two groups based on the mean serum APN level on admission. The data on coronary angiograms and laboratory examinations were collected and compared between groups. The incidence of major adverse cardiac events (MACE) was evaluated in all enrolled patients.

**Results:**

The prevalence of Thrombolysis In Myocardial Infarction (TIMI) flow grade <3 after PPCI and corrected TIMI frame count were lower in the high‐APN group (*P* = 0.032 and *P* = 0.029, respectively). Logistic regression analysis demonstrated that APN was an independent negative predictor of poor coronary flow after PPCI (odds ratio = 0.72, 95% CI: 0.56‐0.93, *P* = 0.011). Kaplan‐Meier curves showed that a higher APN level correlated with a better MACE‐free survival rate, and multivariate Cox hazard regression analysis indicated that high APN was a significant negative predictor of MACE (hazard ratio = 0.54, 95% CI: 0.29‐1.00,* P* = 0.048).

**Conclusion:**

Elevated serum levels of APN on admission are associated with improved myocardial blood flow and clinical outcomes in STEMI patients treated with PPCI.

## 1 INTRODUCTION

Adiponectin (APN), a type of adipocytokine, is secreted from the adipose tissue. Previous studies have demonstrated a significant association of hypoadiponectinemia with the occurrence of acute myocardial infarction (AMI).^1^, ^2^ But the relationship between plasma APN level and prognosis of AMI remains controversial.^3^, ^4^, ^5^, ^6^, ^7^, ^8^, ^9^ Several clinical trials have suggested an inverse correlation of circulating APN level with adverse outcomes in patients with AMI.^7^, ^8^, ^9^ However, the results of other studies are contradictory.^3^, ^4^, ^5^, ^6^ Animal studies showed that myocardial ischemia/reperfusion (MI/R) injury was severe in the APN knockout mice, which was ameliorated by administering exogenous APN.^10^, ^11^ Also, APN could improve vascular endothelial function via its ability to increase nitric oxide production.^12^, ^13^ However, it is still unclear whether APN could protect the cardiomyocytes and improve coronary flow in patients with ST‐segment elevation myocardial infarction (STEMI) treated with primary percutaneous coronary intervention (PPCI).

The no‐reflow/slow‐flow phenomenon is common in patients with STEMI after reperfusion therapies, and it is associated with major adverse cardiac events (MACE).^14^, ^15^ The pathophysiology of no reflow or slow flow involves microembolization, microvascular spasm, microcirculation disturbance, and MI/R injury.^16^ The cardioprotective effect of APN might be related to good myocardial perfusion and clinical outcomes in patients treated with PPCI.

The present study investigated the association between APN on admission and the related indicators of myocardial damage and coronary blood flow in STEMI patients undergoing PPCI. We also aimed to observe the relationship between APN and prognosis in these patients.

## 2 MATERIALS AND METHODS

### 2.1 Participants

A total of 144 patients (114 males and 30 females aged 61.2 ± 12.8 years) with STEMI admitted at Beijing Friendship Hospital from March 2014 to March 2015 were enrolled in this study. The inclusion criteria were STEMI within 12 hours after the onset of symptoms and receiving emergency coronary angiography and PPCI. The exclusion criteria were serious diseases affecting prognosis (renal and hepatic failure, severe anemia, cancer, and acute infectious and inflammatory diseases), active hemorrhage, autoimmune diseases, significant valvular diseases, and AMI with severe stenosis unsuitable for PCI or without significant stenosis. This protocol was approved by the Ethical Committee of Beijing Friendship Hospital, and all patients provided written informed consent.

### 2.2 Coronary angiography and PPCI

All participants received the standard therapies of STEMI before and after PPCI. Once a diagnosis of STEMI was confirmed, PPCI was performed immediately by experienced interventional cardiologists after the administration of 300 mg aspirin and 600 mg clopidogrel as loading doses. At least one drug‐eluting stent was implanted in the culprit lesion of infarct‐related artery (IRA). Dual antiplatelet therapy (100 mg aspirin and 75 mg clopidogrel per day) was continued for 12 months, and then, single antiplatelet therapy (100 mg aspirin per day) was maintained.

Coronary angiograms were interpreted by two independent cardiologists in the catheterization laboratory. Thrombolysis In Myocardial Infarction (TIMI) flow grade (TFG) 3 was defined as complete coronary flow within three cardiac cycles, whereas TFG <3 denoted incomplete perfusion or complete perfusion over three cardiac cycles. The corrected TIMI frame count (CTFC) was calculated using the method previously described in which the frames were counted according to the contrast that flowed from the ostium of the target vessel to the distal landmark branch.^17^ In addition, the CTFC of the left anterior descending artery was adjusted by dividing by 1.7.

### 2.3 Blood sampling

Venous blood samples were obtained from peripheral veins before the PPCI procedure. The serum APN level was determined using the enzyme‐linked immunosorbent assay, and other biomarkers, such as interleukin‐6 (IL‐6), leptin, and high‐sensitive C‐reactive protein (Hs‐CRP), were measured simultaneously. The results of laboratory examinations, including routine blood parameters, renal function, uric acid, lipid, glucose, albumin, creatine kinase (CK)‐MB, and N‐terminal pro‐brain natriuretic peptide (NT‐proBNP), were also compared in this study.

### 2.4 Clinical follow‐up

Major adverse cardiac events, defined as death, nonfatal reinfarction, and requirement of hospitalization for unstable angina pectoris (UAP) or decompensated heart failure (HF), were observed during the follow‐up period that lasted until March 2018. All patients were followed up for at least 3 years or until an endpoint occurred. The follow‐up data were collected from the medical records, patients, and/or their families by telephone interviews and/or outpatient visits.

### 2.5 Statistical analysis

Normally distributed continuous variables were expressed as mean ± SD, whereas those with non‐Gaussian distribution were presented as median with interquartile interval. Categorical variables were recorded with counts and percentages. Baseline characteristics, concomitant medications, and observational indicators were compared using the Student's *t* test or Mann‐Whitney *U* test for continuous variables and using the chi‐square or Fisher's exact test for categorical variables. The predictors of poor coronary flow after PPCI were assessed using the multivariate logistic regression analysis including variables with *P < *0.15 in univariate analysis. Results were expressed as odds ratio (OR) and confidence interval (CI). Univariate and multivariate Cox proportional hazards regression analyses were used to find out the factors associated with MACE by calculating hazard ratios (HRs) and 95% CI. All variables with* P < *0.15 on the univariate analysis were entered into the multivariate model using the step‐wise backward selection method, and a *P* value <0.05 was set for inclusion in the multivariate model. The log‐rank test for Kaplan‐Meier survival curves was used to evaluate the difference in MACE‐free survival time between groups. A *P* value <0.05 was considered statistically significant (2‐sided). All statistical analyses were performed with SPSS 26.0 software (IBM, Armonk, NY, USA).

## 3 RESULTS

### 3.1 Comparison of baseline characteristics

In this study, data of APN on admission displayed a normal distribution, and the mean serum APN level was 13.56 ± 2.14 μg/mL. The patients were divided into two groups based on the average APN level on admission: low‐APN group (*n* = 77, APN <13.56 μg/mL) and high‐APN group (*n* = 67, APN ≥13.56 μg/mL).

The baseline clinical characteristics and concomitant medications of the two groups are shown in Table 1. No significant differences were found in baseline characteristics, including age, sex, coronary risk factors, obesity indexes, blood pressure, and heart rate on admission, between groups. The prevalences of HF and anterior myocardial infarction, as well as the time of symptom to admission and door to balloon, were similar in the two groups. All patients received dual antiplatelet therapy and other conventional medications according to the individual condition. No differences in concomitant medications were observed between the two groups.

**Table 1:** Baseline clinical characteristics and concomitant medications

Clinical features	Low‐APN group (n = 77)	High‐APN group (n = 67)	*P *value
Age, y	61.6 ± 13.3	60.7 ± 12.2	0.672
Male, n (%)	57 (74.0)	57 (85.1)	0.103
Hypertension, n (%)	47 (61.0)	38 (56.7)	0.599
Diabetes, n (%)	18 (23.4)	20 (29.9)	0.379
Dyslipidemia, n (%)	39 (50.6)	33 (49.3)	0.867
Smoking, n (%)	47 (61.0)	50 (74.6)	0.083
Family history of CHD, n (%)	11 (14.3)	12 (17.9)	0.554
BMI, kg/m^2^	25.8 ± 3.3	25.4 ± 3.5	0.438
Waist circumference, cm	89.2 ± 8.1	87.3 ± 8.2	0.162
Heart rate, bpm	73.5 ± 13.0	73.7 ± 14.0	0.932
Systolic blood pressure, mm Hg	120.3 ± 23.6	122.4 ± 25.2	0.607
Diastolic blood pressure, mm Hg	72.8 ± 13.4	73.3 ± 15.3	0.833
Killip class II‐IV, n (%)	19 (24.7)	18 (26.9)	0.764
LVEF, %	56.2 ± 8.1	58.5 ± 8.3	0.097
Anterior MI, n (%)	36 (46.8)	28 (41.8)	0.550
Onset‐to‐admission time, min	210 (165‐330)	240 (180‐360)	0.540
Door‐to‐balloon time, min	90 (75‐110)	85 (70‐100)	0.374
Tirofiban, n (%)	59 (76.6)	48 (71.6)	0.495
ACEI/ARB, n (%)	65 (84.4)	51 (76.1)	0.210
β‐Blocker, n (%)	60 (77.9)	56 (83.6)	0.392
Statin, n (%)	77 (100.0)	67 (100.0)	NS
CCB, n (%)	21 (27.3)	13 (19.4)	0.218

ACEI, angiotensin‐converting enzyme inhibitor; APN, adiponectin; ARB, angiotensin II receptor blocker; BMI, body mass index; bpm, beat per minute; CCB, calcium channel blocker; CHD, coronary artery disease; LVEF, left ventricular ejection fraction; MI, myocardial infarction.

### 3.2 Comparison of laboratory parameters and circulating biomarkers

The laboratory parameters and circulating biomarkers were measured and compared; the results are shown in Table 2. The levels of peak CK‐MB and IL‐6 on admission were significantly lower in the high‐APN group compared with the low‐APN group (*P = *0.032 and *P = *0.003, respectively). In addition, no significant differences were observed in other laboratory indicators between groups, including Hs‐CRP, leptin, and peak NT‐proBNP.

**Table 2:** Baseline laboratory parameters and biomarkers

Variables	Low‐APN group (n = 77)	High‐APN group (n = 67)	*P* value
WBC count, ×10^9^/L	9.69 ± 2.72	10.03 ± 2.81	0.466
Hemoglobin, g/L	138.43 ± 16.83	134.07 ± 17.33	0.129
Platelet count, ×10^9^/L	230.03 ± 62.89	216.04 ± 54.51	0.159
Creatinine, µmol/L	76.04 ± 16.92	81.48 ± 22.49	0.101
Blood urea nitrogen, mmol/L	5.71 ± 1.91	5.79 ± 1.74	0.786
eGFR, mL/min per 1.73 m^2^	94.57 ± 25.52	90.75 ± 28.53	0.398
Uric acid, µmol/L	321.56 ± 74.39	331.22 ± 98.55	0.513
Total cholesterol, mmol/L	4.33 ± 0.87	4.26 ± 1.06	0.669
Triglycerides, mmol/L	1.54 ± 0.81	1.72 ± 1.07	0.236
HDL cholesterol, mmol/L	1.15 ± 0.25	1.08 ± 0.24	0.078
LDL cholesterol, mmol/L	2.50 ± 0.69	2.55 ± 0.87	0.698
Albumin, g/L	41.05 ± 3.68	41.31 ± 4.21	0.696
Admission blood glucose, mmol/L	9.28 ± 3.54	10.18 ± 5.13	0.227
Hemoglobin A1c, %	5.90 (5.50‐6.90)	5.80 (5.40‐6.75)	0.564
Peak CK‐MB, ng/mL	122.00 (57.30‐261.00)	88.40 (45.90‐181.60)	0.037
Peak NT‐proBNP, pg/mL	2033 (924‐4883)	1696 (696‐4299)	0.437
Hs‐CRP, mg/L	7.24 (3.19‐14.38)	4.00 (1.71‐14.79)	0.226
IL‐6, pg/mL	53.37 (33.32‐89.60)	36.25 (22.65‐60.92)	0.003
Leptin, pg/mL	7.19 ± 0.95	7.28 ± 0.98	0.588

APN, adiponectin; CK‐MB, MB fraction of creatine kinase; eGFR, estimated glomerular filtration rate; HDL, high‐density lipoprotein; Hs‐CRP, high‐sensitive C‐reactive protein; IL‐6, interleukin‐6; LDL, low‐density lipoprotein; NT‐proBNP, N‐terminal pro‐brain natriuretic peptide; WBC, white blood cell.

### 3.3 Comparison of coronary imaging characteristics

Table 3 summarizes the angiographic characteristics of the two groups. All patients enrolled in this study underwent PPCI, and the mean number of stents implanted in IRA was 1.15 ± 0.49. No significant differences were noted in the location of IRA and the severity of coronary artery disease between groups. Neither the proportions of using single stent and thrombus aspiration nor the prevalence of initial TFG 0 was obviously different in the two groups. In the high‐APN group, the prevalence of TFG 3 after the procedure was higher (*P = *0.032), whereas the CTFC after PPCI was lower (*P = *0.029), compared with the low‐APN group.

**Table 3:** Coronary angiographic profile and PCI procedure

Characteristics	Low‐APN group (n = 77)	High‐APN group (n = 67)	*P* value
Infarct‐related artery			
LAD, n (%)	39 (50.6)	31 (46.3)	0.426
LCX, n (%)	12 (15.6)	7 (10.4)	
RCA, n (%)	26 (33.8)	29 (43.3)	
Multivessel disease, n (%)	63 (81.8)	56 (83.6)	0.780
Gensini score	77.7 ± 36.0	72.3 ± 33.5	0.348
Single stent implantation, n (%)	68 (88.3)	61 (91.0)	0.592
Using thrombus aspiration devices, n (%)	25 (32.5)	22 (32.8)	0.963
Initial TFG 0, n (%)	63 (81.8)	54 (80.6)	0.851
TFG 3 after PCI, n (%)	60 (77.9)	61 (91.0)	0.032
CTFC after PCI, n (%)	36.1 ± 16.9	30.8 ± 11.8	0.029

APN, adiponectin; CTFC, corrected TIMI frame count; LAD, left anterior descending coronary artery; LCX, left circumflex coronary artery; PCI, percutaneous coronary intervention; RCA, right coronary artery; TFG, TIMI flow grade.

### 3.4 Predictor of poor coronary flow after PPCI

As shown in Table 4, variables that might affect the coronary flow were brought into the logistic regression analyses. Through the univariate regression analysis, Gensini score, door‐to‐balloon time ≥90 minutes, and APN on admission were entered into the multivariate model. The final model of multivariable regression analysis demonstrated that APN was an independent negative predictor of poor coronary flow after reperfusion therapy (OR = 0.72, 95% CI: 0.56‐0.93, *P = *0.011).

**Table 4:** Univariate and multivariate logistic regression analyses for the predictors of poor coronary flow after PPCI

Variables	Univariate analysis			Multivariate analysis		
	OR	95% CI	*P *value	OR	95% CI	*P *value
Age	1.01	0.97‐1.04	0.752			
Male sex	0.54	0.20‐1.46	0.221			
Hypertension	0.89	0.36‐2.18	0.790			
Diabetes	0.98	0.36‐2.71	0.971			
Gensini score	1.01	1.00‐1.02	0.103	1.01	0.99‐1.02	0.308
eGFR	1.00	0.99‐1.02	0.810			
Door‐to‐balloon time ≥90 min	2.11	0.83‐5.33	0.116	0.92	0.36‐2.35	0.866
Killip class II‐IV	0.56	0.18‐1.77	0.325			
Initial TFG 0	0.80	0.27‐2.39	0.689			
APN	0.70	0.54‐0.90	0.006	0.72	0.56‐0.93	0.011

APN, adiponectin; CI, confidence interval; eGFR, estimated glomerular filtration rate; OR, odds ratio; PPCI, primary percutaneous coronary intervention; TFG, TIMI flow grade.

### 3.5 MACE

**Figure 1:**
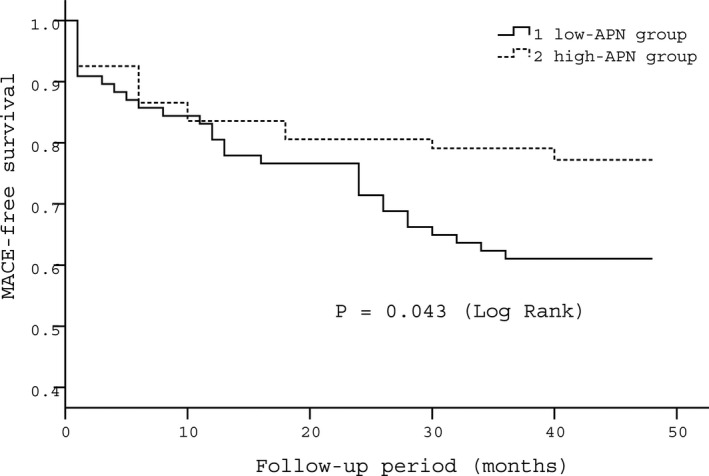
Kaplan‐Meier curves for MACE‐free survival of the two groups. APN, adiponectin; MACE, major adverse cardiac events

The median follow‐up time was 41 months (interquartile range 39‐45). Altogether, eight patients, four in each group, died in the 3‐year follow‐up period. Of these, two patients died during hospitalization. No significant differences were observed in the rates of death and nonfatal reinfarction between groups. However, the incidences of rehospitalization for UAP or HF and total MACE were significantly lower in the high‐APN group than those in the low‐APN group during the 3‐year follow‐up (*P = *0.017 and *P = *0.019, respectively; Table 5). Kaplan‐Meier curves indicated that the patients in the high‐APN group had a better MACE‐free survival rate (*P* = 0.043; Figure 1).

**Table 5:** Clinical outcomes of 3‐y follow‐up

Variables	Low‐APN group (n = 77)	High‐APN group (n = 67)	*P *value
Death, n (%)	4 (5.2)	4 (6.0)	1.000
Nonfatal reinfarction, n (%)	6 (7.8)	3 (4.5)	0.635
Rehospitalization for UAP or HF, n (%)	20 (26.0)	7 (10.4)	0.017
Total MACE, n (%)	30 (39.0)	14 (20.9)	0.019

APN, adiponectin; HF, heart failure; MACE, major adverse cardiac events; UAP, unstable angina pectoris.

### 3.6 Predictors of MACE

Table 6 shows the results of univariate and multivariate Cox hazard regression analyses for factors associated with MACE. APN and conventional risk factors (age, gender, hypertension, diabetes, estimated glomerular filtration rate, door‐to‐balloon time, Killip class, multivessel disease, and TFG) were assessed together. Multivariate Cox hazard regression analysis indicated that the increased serum APN level was a negative predictor of MACE (HR = 0.54, 95% CI: 0.29‐1.00, *P* = 0.048), whereas age ≥70 years positively correlated with MACE (HR = 2.23, 95% CI: 1.23‐4.06, *P = *0.008).

**Table 6:** Univariate and multivariate Cox proportional hazards analyses of data for factors associated with MACE

Variables	Univariate analysis			Multivariate analysis		
	HR	95% CI	*P *value	HR	95% CI	*P *value
Age ≥70 y	2.22	1.22‐4.03	0.009	2.23	1.23‐4.06	0.008
Male sex	1.24	0.63‐2.44	0.538			
Hypertension	1.05	0.58‐1.90	0.877			
Diabetes	0.65	0.31‐1.36	0.253			
eGFR <60 mL/min per 1.73 m^2^	2.27	1.05‐4.87	0.036		Not selected^a^	
Door‐to‐balloon time>90 min	0.88	0.49‐1.59	0.679			
Killip class II‐IV	1.82	1.00‐3.33	0.052		Not selected^a^	
Multivessel disease	1.27	0.55‐2.77	0.607			
TFG <3	0.77	0.33‐1.82	0.548			
High APN (≥13.56 μg/mL)	0.54	0.29‐1.00	0.050	0.54	0.29‐1.00	0.048

APN, adiponectin; CI, confidence interval; eGFR, estimated glomerular filtration rate; HR, hazard ratio; MACE, major adverse cardiac events; TFG, TIMI flow grade.

a This variable was not selected in the step‐wise backward selection entry method.

## 4 DISCUSSION

ST‐segment elevation myocardial infarction is a severe cardiovascular disease causing high mortality.^18^, ^19^, ^20^ In addition, patients with STEMI tend to suffer from HF.^21^ However, currently available therapies cannot effectively improve the prognosis of STEMI, despite an increase in the use of PPCI.^18^, ^22^ A retrospective analysis of hospital data in China from 2001 to 2011 revealed that the in‐hospital mortality of STEMI did not decrease while the intensity of testing and treatment increased.^18^ Further efforts are needed to improve the clinical outcomes of patients with STEMI.

Better coronary flow and less myocardial necrosis are associated with better clinical outcomes for patients with STEMI. This study showed that the elevated APN level on admission was related to improved myocardial reperfusion evaluated by TFG and CTFC. And we found that APN was an independent negative predictor of poor coronary flow after PPCI. Experimental data confirmed the cardiovascular protective effects of APN,^23^, ^24^, ^25^ which could stabilize artery plaques and protect against myocardial damage.^26^, ^27^, ^28^ Several studies demonstrated that APN could also reduce MI/R injury and improve coronary blood flow in both animal models and humans.^10^, ^11^, ^24^, ^25^, ^26^ Trifunovic et al^29^ reported that the plasma APN level on admission significantly positively correlated with coronary flow reserve, indicating that the low‐APN level would lead to coronary microcirculatory impairment. Based on these protective effects of APN, the hypothesis that APN might improve myocardial blood flow after stent implantation in patients with STEMI was proposed. Since treatments for the no‐reflow phenomenon are not always efficacious, extraneous APN administration is expected to be a feasible approach.

In this study, the concentrations of peak CK‐MB and IL‐6 significantly decreased in the high‐APN group. It was reported that the levels of peak CK‐MB and IL‐6 positively correlated with the myocardial infarct size.^30^, ^31^ In addition, increased CK‐MB and IL‐6 levels represented poor clinical outcomes in STEMI patients after reperfusion therapies.^34^, ^35^ Natsukawa et al^37^ showed that the serum APN level on admission was significantly associated with the serum area under the curve of CK‐MB. Also, Radwan et al^38^ reported that APN negatively correlated with IL‐6. Moreover, animal experiments demonstrated that intracoronary administration of APN could reduce the extent of myocardial infarction.^26^, ^39^ Therefore, the elevated APN level might lead to a reduction of infarct sizes and adverse cardiovascular events.

The relationship between APN and the severity of coronary artery disease is still unclear, especially in patients with AMI. A few studies showed that the reduced APN level was associated with severe coronary stenosis and high atherosclerotic burden.^40^, ^41^ However, their findings were not confirmed in other studies.^42^, ^43^ Similarly, no obvious correlation between the APN level and the severity of coronary lesions was seen in our research by measuring the Gensini score and the prevalence of multivessel disease. It was suggested that the role of APN in predicting the severity of lesions in STEMI patients might be insignificant.

Lindberg et al^4^ revealed that increased plasma APN could predict all‐cause and cardiovascular mortality in patients with STEMI undergoing PPCI. The present study did not support their findings. No significant difference was observed in the death rate of STEMI patients grouped by the serum APN level, probably on account of the small sample and low mortality. Contrary to the results of some other studies,^3^, ^4^ elevated serum APN levels seemed to reduce the risk of recurrent cardiovascular events. Despite no statistically significant difference in the incidence of death and reinfarction between groups, the prevalences of worsened angina and decompensated HF decreased in the high‐APN group. This was probably because low plasma APN level was associated with vulnerable plaques and left ventricular remodeling.^44^, ^45^ After adjustment for the conventional risk factors by Cox regression analysis, the increased serum APN level on admission was identified as an independent protective factor in patients with STEMI. It might be attributed to the cardioprotective effects of APN, especially the effects of improving coronary blood flow and reducing myocardial infarct area as mentioned earlier.

The present study had several limitations. Firstly, it was a single‐center and small‐sample study, which did not exclude all the interference factors. Therefore, larger and more rigorous clinical trials are required to support the findings. Secondly, despite a decrease in total MACE in the high‐APN group, no significant differences were found in hard endpoints including all‐cause death and nonfatal reinfarction. The relationship between APN and clinical outcomes in patients with STEMI is still uncertain due to the inconsistency in previous findings. Thirdly, this study found a relationship between serum APN level and myocardial blood flow after PPCI, but the mechanism was not fully explained. The pathophysiology of coronary flow regulated by APN needs further investigation.

In conclusion, a higher serum APN level on admission correlated with better myocardial blood flow and less myocardial damage in patients with STEMI undergoing PPCI. Also, the increased serum APN level could predict good clinical outcomes in such populations.

## References

[1] Pischon T , Girman CJ , Hotamisligil GS , Rifai N , Hu FB , Rimm EB . Plasma adiponectin levels and risk of myocardial infarction in men. JAMA. 2004;291:1730‐1737. 15082700 10.1001/jama.291.14.1730

[2] Laughlin GA , Barrett‐Connor E , May S , Langenberg C . Association of adiponectin with coronary heart disease and mortality. The Rancho Bernardo study. Am J Epidemiol. 2007;165:164‐174. 17101706 10.1093/aje/kwk001 PMC2642645

[3] Wilson SR , Sabatine MS , Wiviott SD , et al. Assessment of adiponectin and the risk of recurrent cardiovascular events in patients presenting with an acute coronary syndrome: observations from the Pravastatin Or atorVastatin Evaluation and Infection Trial‐Thrombolysis in Myocardial Infarction 22 (PROVE IT‐TIMI 22). Am Heart J. 2011;161:1147‐1155. 21641362 10.1016/j.ahj.2011.02.014

[4] Lindberg S , Pedersen SH , Møgelvang R , et al. Usefulness of adiponectin as a predictor of all cause mortality in patients with ST‐segment elevation myocardial infarction treated with primary percutaneous coronary intervention. Am J Cardiol. 2012;109:492‐496. 22105783 10.1016/j.amjcard.2011.09.041

[5] Ritsinger V , Brismar K , Malmberg K , et al. Elevated levels of adipokines predict outcome after acute myocardial infarction: A long‐term follow‐up of the Glucose Tolerance in Patients with Acute M1yocardial Infarction cohort. Diab Vasc Dis Res. 2017;14:77‐87. 28185529 10.1177/1479164116678156

[6] Oliveira GB , França JÍ , Piegas LS . Serum adiponectin and cardiometabolic risk in patients with acute coronary syndromes. Arq Bras Cardiol. 2013;101:399‐409. 24029961 10.5935/abc.20130186 PMC4081163

[7] Huang SS , Huang PH , Chen YH , Chiang KH , Chen JW , Lin SJ . Association of adiponectin with future cardiovascular events in patients after acute myocardial infarction. J Atheroscler Thromb. 2010;17:295‐303. 20185863 10.5551/jat.3533

[8] Kojima S , Funahashi T , Otsuka F , et al. Future adverse cardiac events can be predicted by persistently low plasma adiponectin concentrations in men and marked reductions of adiponectin in women after acute myocardial infarction. Atherosclerosis. 2007;194:204‐213. 16970953 10.1016/j.atherosclerosis.2006.07.028

[9] Mittal A , Gupta MD , Meennahalli Palleda G , Vyas A , Tyagi S . Relationship of plasma adiponectin levels with acute coronary syndromes and coronary lesion severity in north Indian population. ISRN Cardiol. 2013;2013:854815. 24386574 10.1155/2013/854815 PMC3872405

[10] Shibata R , Sato K , Pimentel DR , et al. Adiponectin protects against myocardial ischemia‐reperfusion injury through AMPK‐ and COX‐2‐dependent mechanisms. Nat Med. 2005;11:1096‐1103. 16155579 10.1038/nm1295 PMC2828682

[11] Tao L , Gao E , Jiao X , et al. Adiponectin cardioprotection after myocardial ischemia/reperfusion involves the reduction of oxidative/nitrative stress. Circulation. 2007;115:1408‐1416. 17339545 10.1161/CIRCULATIONAHA.106.666941

[12] Hattori Y , Suzuki M , Hattori S , Kasai K . Globular adiponectin upregulates nitric oxide production in vascular endothelial cells. Diabetologia. 2003;46:1543‐1549. 14551684 10.1007/s00125-003-1224-3

[13] Rojas E , Rodríguez‐Molina D , Bolli P , et al. The role of adiponectin in endothelial dysfunction and hypertension. Curr Hypertens Rep. 2014;16:463. 24924994 10.1007/s11906-014-0463-7

[14] Morishima I , Sone T , Okumura K , et al. Angiographic no‐reflow phenomenon as a predictor of adverse long‐term outcome in patients treated with percutaneous transluminal coronary angioplasty for first acute myocardial infarction. J Am Coll Cardiol. 2000;36:1202‐1209. 11028471 10.1016/s0735-1097(00)00865-2

[15] Harrison RW , Aggarwal A , Ou FS , et al. Incidence and outcomes of no‐reflow phenomenon during percutaneous coronary intervention among patients with acute myocardial infarction. Am J Cardiol. 2013;111:178‐184. 23111142 10.1016/j.amjcard.2012.09.015

[16] Jaffe R , Charron T , Puley G , Dick A , Strauss BH . Microvascular obstruction and the no‐reflow phenomenon after percutaneous coronary intervention. Circulation. 2008;117:3152‐3156. 18559715 10.1161/CIRCULATIONAHA.107.742312

[17] Ito H . The no‐reflow phenomenon associated with percutaneous coronary intervention: its mechanisms and treatment. Cardiovasc Interv Ther. 2011;26:2‐11. 24122492 10.1007/s12928-010-0034-z

[18] Li J , Li X , Wang Q , et al. ST‐segment elevation myocardial infarction in China from 2001 to 2011 (the China PEACE‐Retrospective Acute Myocardial Infarction Study): a retrospective analysis of hospital data. Lancet. 2015;385:441‐451. 24969506 10.1016/S0140-6736(14)60921-1 PMC4415374

[19] Garberich RF , Traverse JH , Claussen MT , et al. ST‐elevation myocardial infarction diagnosed after hospital admission. Circulation. 2014;129:1225‐1232. 24389237 10.1161/CIRCULATIONAHA.113.005568

[20] Pedersen F , Butrymovich V , Kelbæk H , et al. Short‐ and long‐term cause of death in patients treated with primary PCI for STEMI. J Am Coll Cardiol. 2014;64:2101‐2108. 25457398 10.1016/j.jacc.2014.08.037

[21] Wu AH , Parsons L , Every NR , Bates ER ; Second National Registry of Myocardial Infarction . Hospital outcomes in patients presenting with congestive heart failure complicating acute myocardial infarction: a report from the Second National Registry of Myocardial Infarction (NRMI‐2). J Am Coll Cardiol. 2002;40:1389‐1394. 12392826 10.1016/s0735-1097(02)02173-3

[22] Prasad A , Stone GW , Holmes DR , Gersh B . Reperfusion injury, microvascular dysfunction, and cardioprotection: the “dark side” of reperfusion. Circulation. 2009;120:2105‐2112. 19933949 10.1161/CIRCULATIONAHA.108.814640

[23] Villarreal‐Molina MT , Antuna‐Puente B . Adiponectin: anti‐inflammatory and cardioprotective effects. Biochimie. 2012;94:2143‐2149. 22796520 10.1016/j.biochi.2012.06.030

[24] Gonon AT , Widegren U , Bulhak A , et al. Adiponectin protects against myocardial ischaemia‐reperfusion injury via AMP‐activated protein kinase, Akt, and nitric oxide. Cardiovasc Res. 2008;78:116‐122. 18222959 10.1093/cvr/cvn017

[25] Shibata R , Ouchi N , Walsh K , Murohara T . Potential of adiponectin as a cardioprotective agent. Future Cardiol. 2007;3:647‐656. 19804285 10.2217/14796678.3.6.647

[26] Kondo K , Shibata R , Unno K , et al. Impact of a single intracoronary administration of adiponectin on myocardial ischemia/reperfusion injury in a pig model. Circ Cardiovasc Interv. 2010;3:166‐173. 20332381 10.1161/CIRCINTERVENTIONS.109.872044 PMC3668696

[27] Ohashi K , Ouchi N , Matsuzawa Y . Anti‐inflammatory and anti‐atherogenic properties of adiponectin. Biochimie. 2012;94:2137‐2142. 22713764 10.1016/j.biochi.2012.06.008

[28] Zhu W , Cheng KK , Vanhoutte PM , Lam KS , Xu A . Vascular effects of adiponectin: molecular mechanisms and potential therapeutic intervention. Clin Sci (Lond). 2008;114:361‐374. 18230060 10.1042/CS20070347

[29] Trifunovic D , Stankovic S , Marinkovic J , et al. Time‐dependent changes of plasma adiponectin concentration in relation to coronary microcirculatory function in patients with acute myocardial infarction treated by primary percutaneous coronary intervention. J Cardiol. 2015;65:208‐215. 25012060 10.1016/j.jjcc.2014.05.011

[30] Dohi T , Maehara A , Brener SJ , et al. Utility of peak creatine kinase‐MB measurements in predicting myocardial infarct size, left ventricular dysfunction, and outcome after first anterior wall acute myocardial infarction (from the INFUSE‐AMI trial). Am J Cardiol. 2015;115:563‐570. 10.1016/j.amjcard.2014.12.008 25586335

[31] Fiolet JW , ter Welle HF , van Capelle FJ , Lie KI . Infarct size estimation from serial CK MB determinations: peak activity and predictability. Br Heart J. 1983;49:373‐380. 6830671 10.1136/hrt.49.4.373 PMC481316

[32] Puhakka M , Magga J , Hietakorpi S , et al. Interleukin‐6 and tumor necrosis factor alpha in relation to myocardial infarct size and collagen formation. J Card Fail. 2003;9:325‐332. 13680554 10.1054/jcaf.2003.38

[33] Ritschel VN , Seljeflot I , Arnesen H , et al. IL‐6 signalling in patients with acute ST‐elevation myocardial infarction. Results Immunol. 2013;4:8‐13. 24707455 10.1016/j.rinim.2013.11.002 PMC3973821

[34] Bagai A , Schulte PJ , Granger CB , et al. Prognostic implications of creatine kinase‐MB measurements in ST‐segment elevation myocardial infarction patients treated with primary percutaneous coronary intervention. Am Heart J. 2014;168:503‐511. 25262260 10.1016/j.ahj.2014.06.008

[35] Nienhuis MB , Ottervanger JP , de Boer MJ , et al. Prognostic importance of creatine kinase and creatine kinase‐MB after primary percutaneous coronary intervention for ST‐elevation myocardial infarction. Am Heart J. 2008;155:673‐679. 18371475 10.1016/j.ahj.2007.11.004

[36] Fanola CL , Morrow DA , Cannon CP , et al. Interleukin‐6 and the risk of adverse outcomes in patients after an acute coronary syndrome: Observations from the SOLID‐TIMI 52 (stabilization of plaque using darapladib‐thrombolysis in myocardial infarction 52) trial. J Am Heart Assoc. 2017;6:e005637. 29066436 10.1161/JAHA.117.005637 PMC5721825

[37] Natsukawa T , Maeda N , Fukuda S , et al. Significant association of serum adiponectin and creatine kinase‐MB levels in ST‐segment elevation myocardial infarction. J Atheroscler Thromb. 2017;24:793‐803. 28100880 10.5551/jat.38232 PMC5556187

[38] Radwan DA , Al‐Tahhan MA , Hussein AG , Said H , Kadry YA . Adiponectin and some inflammatory and endothelial markers in type‐2 diabetes with and without cardiovascular disease. Egypt J Immunol. 2005;12:133‐142. 16734148

[39] Dębiński M , Buszman PP , Milewski K , et al. Intracoronary adiponectin at reperfusion reduces infarct size in a porcine myocardial infarction model. Int J Mol Med. 2011;27:775‐781. 21399860 10.3892/ijmm.2011.643

[40] Hara K , Yamauchi T , Imai Y , et al. Reduced adiponectin level is associated with severity of coronary artery disease. Int Heart J. 2007;48:149‐153. 17409580 10.1536/ihj.48.149

[41] Otsuka F , Sugiyama S , Kojima S , et al. Plasma adiponectin levels are associated with coronary lesion complexity in men with coronary artery disease. J Am Coll Cardiol. 2006;48:1155‐1162. 16978998 10.1016/j.jacc.2006.05.054

[42] Amirzadegan A , Shakarami A , Borumand MA , Davoodi G , Ghaffari‐Marandi N , Jalali A . Correlation between plasma adiponectin levels and the presence and severity of coronary artery disease. J Tehran Heart Cent. 2013;8:140‐145. 24396363 PMC3874373

[43] Ji Q , Lin Y , Liang Z , et al. Chemerin is a novel biomarker of acute coronary syndrome but not of stable angina pectoris. Cardiovasc Diabetol. 2014;13:145. 25367628 10.1186/s12933-014-0145-4 PMC4229596

[44] Sawada T , Shite J , Shinke T , et al. Low plasma adiponectin levels are associated with presence of thin‐cap fibroatheroma in men with stable coronary artery disease. Int J Cardiol. 2010;142:250‐256. 19403182 10.1016/j.ijcard.2008.12.216

[45] Piestrzeniewicz K , Luczak K , Maciejewski M , Drozdz J . Low adiponectin blood concentration predicts left ventricular remodeling after ST‐segment elevation myocardial infarction treated with primary percutaneous coronary intervention. Cardiol J. 2010;17:49‐56. 20104457

